# Supplementary material for: Applying a Random Encounter Model to Estimate the Asiatic Black Bear (Ursus thibetanus) Density from Camera Traps in the Hindu Raj Mountains, Pakistan
Source: Biology (Basel). 2024 May 14;13(5):341. doi: 10.3390/biology13050341 (PMC11117995; doi:10.3390/biology13050341)
Supplement: Supplementary file 1 [file biology-13-00341-s001.zip › biology-2912273-supplementary.pdf]

Section S1

R code for calculating Activity Level

```
# R code to estimate wildlife activity patterns and activity levels from camera

# trap data

# Working directory

# This will open an explorer window for easy browsing

setwd(choose.dir())

# Import the .txt table into R when the data are separated by ";"

# Load data frame

data_activity <- read.table("Data.txt", sep = ";", dec=".", header=TRUE, as.is=TRUE) # data frame

# Data frame structure:

# N_ind: Number of individuals recorded

# Time: Time (HH:MM:SS format) when animals were recorded

#install.packages ("activity") # just in case you didn’t install the package previously

library(activity) # load package

# Replicating each sequence based on the number of animals recorded

data_activity.r <- data_activity[rep(row.names(data_activity), data_activity$N_ind), 1:2]

# Convert time of data to a numeric vector of radian time-of-day

data_activity.r$radtime <- gettime(data_activity.r$Time, "%H:%M:%S", "proportion")

radtime <- 2*pi*gettime(data_activity.r$Time, "%H:%M:%S", "proportion")

# fit activity model

actmod <- fitact(radtime, sample="data")

# plot activity pattern
```

```
plot(actmod)

# activity level

actmod@act

legend('topleft', c("Activity level = 0.41(0.27-0.55), SE = 0.071"), col=c("black", "blue"),
bty='n')

#Add sunrise and sunset time of your study area

abline(v=c(5, (18.5) - 24), col=c("blue"), lty=5)

abline(v=c(7, (17.5) - 24), col=c("blue"), lty=5)

abline(v=c(19, (18.5) - 24), col=c("blue"), lty=5)

abline(v=c(17, (18.5) - 24), col=c("blue"), lty=5)

abline(v=c(6.5, 17+30/60), lty=5)
```

Section S2

Variance associated with encounter rate

```
NONPARAMETRIC BOOTSTRAP


Call:
boot(data = data, statistic = statistic_function, R = 10000)


Bootstrap Statistics :
     original        bias    std. error
t1* 0.1433478 -0.0002126522  0.03199916
> # Estimate the variance of the encounter rate
> bootstrap_variance <- var(bootstrap_results$t)
> cat("Estimated variance of the encounter rate:", bootstrap_variance, "\n")
Estimated variance of the encounter rate: 0.001023946
```

Variance associated with Radius and Angle

```
NONPARAMETRIC BOOTSTRAP


Call:
boot(data = data, statistic = statistic_function_radius, R = 10000)


Bootstrap Statistics:
       original        bias     std. error
t1* 0.004518116 -1.746014e-06 0.0002502456
> # Print the bootstrap results for angle
> print(bootstrap_results_angle)

NONPARAMETRIC BOOTSTRAP


Call:
boot(data = data, statistic = statistic_function_angle, R = 10000)


Bootstrap Statistics :
     original        bias    std. error
t1* 0.4264674 -0.0001045181  0.02925566
> # Estimate and print the variance for the radius
> bootstrap_variance_radius <- var(bootstrap_results_radius$t)
> cat("Estimated variance of the detection radius:", bootstrap_variance_radiu
s, "\n")
Estimated variance of the detection radius: 6.262285e-08
> # Estimate and print the variance for the angle
> bootstrap_variance_angle <- var(bootstrap_results_angle$t)
> cat("Estimated variance of the detection angle:", bootstrap_variance_angle,
 "\n")
Estimated variance of the detection angle: 0.0008558938
```

Variance associated with Speed

```
NONPARAMETRIC BOOTSTRAP


Call:
boot(data = data, statistic = statistic_function_speed, R = 10000)


Bootstrap Statistics :
    original        bias    std. error
t1* 2.174182 0.0002983636   0.2284915
> # Estimate and print the variance for the speed
> bootstrap_variance_speed <- var(bootstrap_results_speed$t)
> cat("Estimated variance of the speed:", bootstrap_variance_speed, "\n")
Estimated variance of the speed: 0.05220835
```

Section S3

Variance associated with day range calculations and Density calculations along with its variance.

Let's use the delta method with partial derivatives to calculate the variance of the day range (*D*) which is a function of the activity level (*a*) and speed (*s*). The day range *D* is given by:

Given:

- Activity level (*a*): mean = 0.41 X 24 = 9.84, SE = 1.706
- Speed (*s*): mean = 2.17, Variance = 0.05
- We can convert the standard errors to variances:

$$Var(a) = \left(SE(a)\right)^2 = (1.706)^2$$

$$\mathrm{D} = \mathrm{a} \times \mathrm{s}$$

The partial derivatives are:

With respect to a:

$$\partial D/\,\partial a = \mathrm{s}$$

With respect to s:

$$\partial D/\,\partial s = a$$

Using the delta method formula for the variance of a function of independent variables:

$$\mathrm{Var(D)} = (\partial D/\partial a\,)^2 Var(a) + (\partial D/\partial s)^2 Var(s)$$

Plugging in the partial derivatives and the variances of a and s:

$$\text{Var(D)} = (\text{s})^2\ \text{Var(a)} + (\text{a})^2\ \text{Var(s)} = (2.17)^2 \times (1.706)^2 + (9.84)^2 \times (0.05)$$
$$= 4.7089 \times 2.910436 + 96.8064 \times 0.051984$$
$$= 13.706484644 + 5.032384256$$

$$\text{Var(D)} = 18.7388689$$

So, the variance of the day range (D) using the delta method is 18.74. To find the standard error of the day range, we would take the square root of the variance:

$$\text{SE}(D) = \sqrt{\text{V}}\text{ar}(D) = \sqrt{1}8.74 = 4.33$$

Day range

V = 21.39 km/day ± 4.33 km/day

Density calculation

- Encounter rate (y/t): 0.1433478.
- Day range (v): 21.39395 km/day.
- Effective radius (r): 0.004518116 km.
- Angle of the camera detection zone (θ): 0.4264674 radians.

$$D = y/t \times \pi/v \times r \times (2 + \theta)$$

Using REM equation:

$$D = 0.1433478 \times \pi/21.39395 \times 0.004518116 \times (2 + 0.4264674)$$

The calculated density (D) is **1.8752** individuals per square kilometer.

Given the values for the parameters:

$$Var(y/t) = 0.001023946$$

$$Var(v) = 18.74\ km^2/day^2$$

$$Var(r) = 6.262285 \times 10^{-8} km^2$$

$$Var(\theta) = 0.0008558938\ radians^2$$

- **Encounter rate (y/t): 0.004518116.**
- **Day range (v): 21.39 km/day**

- **Radius (*r*): 0.004518116 km**
- **Angle (θ): 0.4264674 radians.**

We can now evaluate the partial derivatives at these values:

With respect to y/t:

$$\partial D \ / \ \partial (y/t) = \pi \ / \ 21.39 \times 0.004518116 \times (2 + 0.4264674) = 5.8107$$

With respect to v:

$$\begin{aligned} \partial D / \ \partial v &= -0.004518116 \times \pi/(21.39)^2 \times 0.004518116 \times (2 + 0.4264674) \\ &= -0.00013539 \end{aligned}$$

With respect to *r:*

$$\partial D / \ \partial r = -0.004518116 \times \pi//21.39 \times (0.004518116)^2 \times (2 + 0.4264674) = -5.8107$$

With respect to *θ:*

$$\partial D / \partial \theta = -0.004518116 \times \pi/21.39 \times 0.004518116 \times (2 + 0.4264674)^2 = -0.009109$$

Now, we can plug in these partial derivatives and the variances into the formula for Var (D)

$$\begin{aligned} Var(D) &= 2 \times 0.001023946 + (-0.00013539)^2 \times 18.74 + (-5.8107)^2 \times 6.262285 \times 10^{-8} \\ &\quad + (-0.009109)^2 \times 0.0008558938 \end{aligned}$$

$$= 0.03453 + 5.30 \times \ 10^{-8} + 2.12 \times 10^{-6} + 7.09 \times 10^{-8}$$

$$Var(D) = 0.03453$$

So, the variance of the density *D* is 0.03453 individuals^2/km^4. The standard error of *D* would be the square root of the variance:

$$SE(D) = \sqrt{0.}03453 = 0.1858 \ individuals/km^2$$

Therefore, the standard error of the density (D) is 0.1858 individuals/km^2.

Section S4

R codes for bootstrapping

```
# Load necessary libraries

library(boot)

library(readr)

# Load necessary libraries

library(boot)

library(readr)

library(dplyr)

# Read the data from the CSV file

data <- read_csv("density2.csv")

# Read the data from the CSV file

data <- read_csv("path/to/your/density2.csv")

# Define the statistic function to calculate the mean encounter rate

statistic_function <- function(data, indices) {

  # Resample the data with replacement

  resampled_data <- data[indices, ]

  # Calculate and return the mean encounter rate

  mean(resampled_data$`encounter rate`)

}

# Perform bootstrapping with 10000 replicates

bootstrap_results <- boot(data, statistic = statistic_function, R = 10000)
```

```
# Print the bootstrap results
print(bootstrap_results)
# Estimate the variance of the encounter rate
bootstrap_variance <- var(bootstrap_results$t)
cat("Estimated variance of the encounter rate:", bootstrap_variance, "\n")m

# Load necessary libraries
library(boot)
library(readr)

# Read the data from the CSV file
data <- read_csv("density.csv")
# Load necessary libraries
library(boot)
library(readr)
library(dplyr)

# Read the data from the CSV file after removing the Speed column
data <- read_csv("/path/to/your/data.csv") %>%
  select(Radius, Angle)  # Select only the columns of interest

# Define a statistic function for the radius
statistic_function_radius <- function(data, indices) {
```

```
  resampled_data <- data[indices, ]
  mean(resampled_data$Radius)  # Replace 'Radius' with the actual column name for radius in your dataset
}

# Define a statistic function for the angle
statistic_function_angle <- function(data, indices) {
  resampled_data <- data[indices, ]
  mean(resampled_data$Angle)  # Replace 'Angle' with the actual column name for angle in your dataset
}

# Perform bootstrapping for the radius
bootstrap_results_radius <- boot(data, statistic = statistic_function_radius, R = 10000)

# Perform bootstrapping for the angle
bootstrap_results_angle <- boot(data, statistic = statistic_function_angle, R = 10000)

# Print the bootstrap results for radius
print(bootstrap_results_radius)

# Print the bootstrap results for angle
print(bootstrap_results_angle)

# Estimate and print the variance for the radius
```

```
bootstrap_variance_radius <- var(bootstrap_results_radius$t)
cat("Estimated variance of the detection radius:", bootstrap_variance_radius, "\n")

# Estimate and print the variance for the angle
bootstrap_variance_angle <- var(bootstrap_results_angle$t)
cat("Estimated variance of the detection angle:", bootstrap_variance_angle, "\n")

  # Load necessary libraries
library(readr)
library(boot)
library(readr)
library(dplyr)

# Read the data from the CSV file
data <- read_csv("speed.csv")

# Read the speed data from the CSV file
data <- read_csv("speed.csv")

# Check the first few rows of the speed data to confirm it's loaded correctly
head(data)

# Define a statistic function for speed
statistic_function_speed <- function(data, indices) {
```

```
  # Resample the data with replacement
  resampled_data <- data[indices, ]
  # Calculate and return the mean speed
  mean(resampled_data$speed)  # Make sure 'speed' matches the column name in your
CSV
}

# Perform bootstrapping for the speed
bootstrap_results_speed <- boot(data, statistic = statistic_function_speed, R = 10000)

# Print the bootstrap results for speed
print(bootstrap_results_speed)

# Estimate and print the variance for the speed
bootstrap_variance_speed <- var(bootstrap_results_speed$t)
cat("Estimated variance of the speed:", bootstrap_variance_speed, "\n")
```

Section S5

Executing: Exploratory Regression

```
Extract_shp2 Encounter rateEUCALIDEAN DISTANCE TO BUILT IN
AREAS;Altitude;Forest_cover;Aspect;Slope;Roughness # # # 6 1 0.3 0.05 7.5 0.1
0.1
Start Time: Fri Apr  5 06:22:25 2024
Running script ExploratoryRegression...
*****************************************************************************
*
Choose 1 of 6 Summary
     Highest Adjusted R-Squared Results
AdjR2  AICc   JB K(BP)  VIF   SA   Model
 0.49 -6.64 0.48  0.22 1.00 0.65  -ALTITUDE***
 0.24  2.24 0.39  0.06 1.00 0.72  -EUCALIDEAN DISTANCE TO BUILT IN AREAS**
 0.13  5.36 0.42  0.01 1.00 0.69  +FOREST_COVER***
                        Passing Models
AdjR2    AICc      JB       K(BP)    VIF      SA         Model
0.485819 -6.635059 0.481256 0.221983 1.000000 0.653428  -ALTITUDE***

*****************************************************************************
*
Choose 2 of 6 Summary
           Highest Adjusted R-Squared Results
AdjR2  AICc   JB K(BP)  VIF   SA   Model
 0.58 -9.48 0.97  0.11 1.00 0.44  -ALTITUDE***  -ASPECT**
 0.57 -8.68 0.50  0.58 1.12 0.61  -EUCALIDEAN DISTANCE TO BUILT IN AREAS**  -
ALTITUDE***
 0.55 -8.01 0.41  0.38 1.01 0.88  -ALTITUDE***  -ROUGHNESS*
                                  Passing Models
AdjR2    AICc      JB       K(BP)    VIF      SA         Model
0.580568 -9.482647 0.968810 0.109898 1.004023 0.440121  -ALTITUDE***  -
ASPECT**
0.565598 -8.676053 0.497510 0.577306 1.121692 0.611096  -EUCALIDEAN DISTANCE
TO BUILT IN AREAS**  -ALTITUDE***
0.360897 0.204113  0.343788 0.184150 1.006418 0.364702  -EUCALIDEAN DISTANCE
TO BUILT IN AREAS***  +FOREST_COVER**

*****************************************************************************
*
Choose 3 of 6 Summary
                   Highest Adjusted R-Squared Results
AdjR2  AICc   JB K(BP)  VIF   SA   Model
 0.63 -9.96 0.74  0.90 1.17 0.25  -EUCALIDEAN DISTANCE TO BUILT IN AREAS**  -
ALTITUDE***  +FOREST_COVER*
 0.62 -9.93 0.53  0.44 1.19 0.41  -EUCALIDEAN DISTANCE TO BUILT IN AREAS*  -
ALTITUDE***  -ASPECT*
 0.62 -9.81 0.19  0.81 1.13 0.62  -EUCALIDEAN DISTANCE TO BUILT IN AREAS**  -
ALTITUDE***  -ROUGHNESS*
       Passing Models
AdjR2 AICc JB K(BP) VIF SA   Model

*****************************************************************************
*
```

```
Choose 4 of 6 Summary
                        Highest Adjusted R-Squared Results
AdjR2  AICc   JB K(BP)  VIF   SA   Model
 0.66 -9.69 0.32  0.97 1.21 0.31  -EUCALIDEAN DISTANCE TO BUILT IN AREAS*  -
ALTITUDE***  +FOREST_COVER  -ASPECT
 0.64 -8.69 0.15  0.91 1.21 0.37  -EUCALIDEAN DISTANCE TO BUILT IN AREAS**  -
ALTITUDE***  +FOREST_COVER  -ROUGHNESS
 0.64 -8.17 0.08  0.76 1.19 0.42  -EUCALIDEAN DISTANCE TO BUILT IN AREAS**  -
ALTITUDE***  +FOREST_COVER*  -SLOPE
       Passing Models
AdjR2 AICc JB K(BP) VIF SA   Model

*****************************************************************************
*
Choose 5 of 6 Summary
                            Highest Adjusted R-Squared Results
AdjR2  AICc   JB K(BP)  VIF   SA   Model
 0.65 -5.98 0.11  0.93 1.64 0.37  -EUCALIDEAN DISTANCE TO BUILT IN AREAS*  -
ALTITUDE***  +FOREST_COVER  -ASPECT  -ROUGHNESS
 0.65 -5.82 0.07  0.80 1.48 0.37  -EUCALIDEAN DISTANCE TO BUILT IN AREAS*  -
ALTITUDE***  +FOREST_COVER  -ASPECT  -SLOPE
 0.62 -4.51 0.10  0.87 3.48 0.39  -EUCALIDEAN DISTANCE TO BUILT IN AREAS**  -
ALTITUDE***  +FOREST_COVER  -SLOPE  -ROUGHNESS
       Passing Models
AdjR2 AICc JB K(BP) VIF SA   Model

*****************************************************************************
*
Choose 6 of 6 Summary
                                Highest Adjusted R-Squared Results
AdjR2  AICc   JB K(BP)  VIF   SA   Model
 0.63 -1.17 0.08  0.89 3.77 0.37  -EUCALIDEAN DISTANCE TO BUILT IN AREAS*  -
ALTITUDE***  +FOREST_COVER  -ASPECT  -SLOPE  -ROUGHNESS
       Passing Models
AdjR2 AICc JB K(BP) VIF SA   Model

*****************************************************************************
*
************* Exploratory Regression Global Summary (ENCOUNTE_1)
*************

              Percentage of Search Criteria Passed
                   Search Criterion Cutoff Trials # Passed % Passed
             Min Adjusted R-Squared > 0.30     63       39    61.90
            Max Coefficient p-value < 0.05     63        6     9.52
                     Max VIF Value < 7.50     63       63   100.00
            Min Jarque-Bera p-value > 0.10     63       59    93.65
Min Spatial Autocorrelation p-value > 0.10     19       19   100.00

-----------------------------------------------------------------------------
-

       Summary of Variable Significance
Variable     % Significant % Negative % Positive
ALTITUDE            100.00     100.00       0.00
EUCALIDEAN DISTANCE TO BUILT IN AREAS           71.88     100.00       0.00
FOREST_COVER         40.62       0.00     100.00
```

```
ASPECT                3.12     100.00       0.00
SLOPE                 0.00      84.38      15.62
ROUGHNESS             0.00      87.50      12.50

-----------------------------------------------------------------------------
-

    Summary of Multicollinearity
Variable      VIF Violations Covariates
EUCALIDEAN DISTANCE TO BUILT IN AREAS   1.20     0      --------
ALTITUDE     1.22     0      --------
FOREST_COVER 1.25     0      --------
ASPECT       1.60     0      --------
SLOPE        3.21     0      --------
ROUGHNESS    3.77     0      --------

-----------------------------------------------------------------------------
-

                           Summary of Residual Normality (JB)
      JB    AdjR2      AICc    K(BP)      VIF       SA   Model
0.968810 0.580568 -9.482647 0.109898 1.004023 0.440121  -ALTITUDE***  -
ASPECT**
0.795632 0.566802 -6.612457 0.168658 1.390896 0.489425  -ALTITUDE***  -ASPECT
-SLOPE
0.755707 0.606242 -8.807984 0.398001 1.100194 0.385144  -ALTITUDE***
+FOREST_COVER  -ASPECT*

-----------------------------------------------------------------------------
-

             Summary of Residual Spatial Autocorrelation (SA)
      SA    AdjR2      AICc       JB    K(BP)      VIF   Model
0.877411 0.552815 -8.009029 0.407752 0.377780 1.005550  -ALTITUDE***  -
ROUGHNESS*
0.719599 0.243809  2.236474 0.391899 0.061031 1.000000  -EUCALIDEAN DISTANCE
TO BUILT IN AREAS**
0.692350 0.133830  5.359599 0.422079 0.013044 1.000000  +FOREST_COVER***

-----------------------------------------------------------------------------
-

Table Abbreviations
AdjR2 Adjusted R-Squared
AICc  Akaike's Information Criterion
JB    Jarque-Bera p-value
K(BP) Koenker (BP) Statistic p-value
VIF   Max Variance Inflation Factor
SA    Global Moran's I p-value
Model Variable sign (+/-)
Model Variable significance (* = 0.10; ** = 0.05; *** = 0.01)

-----------------------------------------------------------------------------
-
Completed script ExploratoryRegression...
Succeeded at Fri Apr  5 06:22:25 2024 (Elapsed Time: 0.44 seconds)
```

The output of the Exploratory Regression analysis in ArcGIS 10.8 provided a comprehensive summary of the relationships between the dependent variable (bears encounter rates) and the explanatory variables (Euclidean distance to built-in areas, altitude, forest cover, aspect, slope, and roughness). Here's a breakdown of the key results:

1. **Model Selection and Significance**:
   - ❖ The highest Adjusted R-Squared value is 0.66, suggesting that the model with Euclidean distance to built-in areas, altitude, forest cover, and aspect explains 66% of the variance in bear encounter rates.
   - ❖ Altitude is significant in all models (100% significant, always negative), indicating a strong and consistent negative relationship with bear encounter rates.
   - ❖ Euclidean distance to built-in areas is significant in 71.88% of the models, also showing a negative relationship with bear encounter rates.
   - ❖ Forest cover is significant in 40.62% of the models, with a positive relationship with bear encounter rates.
   - ❖ Aspect is significant in a small percentage of models (3.12%), with a negative relationship.
   - ❖ Slope and roughness are not significant in any of the models.
2. **Multicollinearity**:
   - ❖ The Variance Inflation Factor (VIF) values for all variables are below the threshold of 7.5, indicating that multicollinearity is not a concern in these models.
3. **Residual Normality and Spatial Autocorrelation**:
   - ❖ The Jarque-Bera (JB) p-values and Global Moran's I (SA) p-values suggest that the residuals are generally normally distributed and do not exhibit significant spatial autocorrelation, which is desirable for a well-specified model.
4. **Best Models**:

- The best models based on Adjusted R-Squared include combinations of altitude, aspect, Euclidean distance to built-in areas, and forest cover.

5. **Recommendations**:

    - Based on these results, altitude appears to be the most important factor negatively associated with bear encounter rates. Efforts to manage bear populations and reduce human-bear conflicts may need to consider the influence of altitude on bear distribution.
    - The significance of Euclidean distance to built-in areas suggests that proximity to human development may also impact bear encounter rates.
    - The positive association with forest cover indicates that bears may prefer low land Quercus Forest areas, which could inform habitat conservation and management strategies.
